# Effect of thyroid hormone replacement treatment on cardiac diastolic function in adult patients with subclinical hypothyroidism: a meta-analysis

**DOI:** 10.3389/fendo.2023.1263861

**Published:** 2023-09-25

**Authors:** Gejing Liu, Man Ren, Yingshi Du, Ruoyu Zhao, Yu Wu, Yongming Liu, Liang Qi

**Affiliations:** ^1^ Department of Geriatrics, First Hospital of Lanzhou University, Lanzhou, China; ^2^ Department of Gastroenterology and Oncology, Gansu Provincial Cancer Hospital, Lanzhou, China; ^3^ Institute of Evidence-Based Medicine, School of Basic Medical Sciences, Lanzhou University, Lanzhou, China; ^4^ Department of Cardiovascular Surgery, First Hospital of Lanzhou University, Lanzhou, China

**Keywords:** diastolic function, subclinical hypothyroidism, meta-analysis, echocardiography, levothyroxine

## Abstract

**Background:**

Although subclinical hypothyroidism (SCH) is related to abnormalities in left ventricular diastolic function, the use of levothyroxine as a regular treatment remains debatable. This meta-analysis aimed to determine whether thyroid hormone replacement therapy affects cardiac diastolic function in patients with SCH as measured by echocardiography.

**Methods:**

This meta-analysis included a search of the EMBASE, PubMed, Web of Science, and Cochrane Library databases from their inception to May 18, 2023, for studies analyzing cardiac morphology and functional changes in patients with SCH before and after thyroid hormone replacement. The outcome measures were cardiac morphology and diastolic and overall cardiac function, as assessed using ultrasound parameters (including ventricular wall thickness, chamber size, mitral wave flow, tissue Doppler, and speckle tracking). The quality of the studies was assessed using the Newcastle–Ottawa Scale. The standard mean differences (MDs) and 95% confidence intervals (CI) were calculated using fixed- or random-effects models.

**Results:**

Seventeen studies met the inclusion criteria. A total of 568 patients participated and completed the follow-up. All studies specifically stated that serum thyrotropin levels returned to normal by the end of the study period. Compared with baseline levels, no significant morphological changes were observed in the heart. In terms of diastolic function, we discovered that the ratios of E-velocity to A-velocity (E/A) had greatly improved after thyroid hormone replacement therapy, whereas the ratios of the mitral inflow E wave to the tissue Doppler e’ wave (E/e’) had not. Global longitudinal strain (GLS) increased significantly after treatment with levothyroxine.

**Conclusion:**

In adult patients with SCH, thyroid hormone supplementation can partially but not completely improve parameters of diastolic function during the observation period. This meta-analysis was performed according to the Preferred Reporting Items for Systematic Reviews and Meta-Analyses 2020 statement, an updated guideline for reporting systematic reviews (11) and was registered with INPLASY (INPLASY202320083).

**Systematic review registration:**

https://inplasy.com/inplasy-2023-2-0083.

## Introduction

Subclinical hypothyroidism (SCH) exists when free thyroxine (FT4) levels are within the defined reference range but thyroid stimulating hormone (TSH) levels are elevated outside the reference range (1). It is a common disorder which affects approximately 10% of the adult population, and around 1 in 3 patients with SCH are asymptomatic (2). Inadequate serum thyroid hormone levels impair cardiac function and may result in multiple cardiovascular risk factors, such as endothelial dysfunction, increased intima-media thickness, increased vascular resistance, and pericardial effusion (3). Additionally, the risk of heart failure increases with higher serum thyrotropin levels, with the incidence increasing as TSH levels increase, particularly above TSH ≥10 mIU/L, even after adjusting for cardiovascular risk factors (4).

Previous studies have demonstrated that patients with SCH had a higher prevalence of left ventricular diastolic dysfunction (LVDD) than controls (5, 6). SCH diastolic dysfunction mainly manifests as an impaired left ventricular (LV) relaxation pattern, such as longer deceleration time, isovolumic relaxation time, and higher LV filling, compared to euthyroidism (7). The potential mechanisms responsible for diastolic dysfunction of the left ventricle in SCH are related to endothelial dysfunction, arterial stiffness, and the inflammatory state and are driven by TSH apoptosis-derived microparticles (8). Although the association between SCH and LV diastolic function is relatively well established, the benefits of thyroid hormone replacement therapy for SCH in improving cardiac diastolic function remain unclear. One study revealed that treatment with levothyroxine reversed diastolic abnormalities in SCH to comparable levels to those in controls (9). However, a randomized controlled trial (RCT) in older adults with mild SCH showed that diastolic heart function did not differ after treatment with levothyroxine compared to a placebo (10). Considering the potential benefits of thyroid hormone supplementation, the risk of overtreatment should also be considered. More substantive evidence of sufficient cardiovascular benefits from thyroid hormone supplementation for patients with SCH is required. This meta-analysis aimed to explore the value of levothyroxine application in terms of cardiac diastolic function in adult patients with SCH to provide a basis for the clinical use of hormone replacement therapy in the SCH population.

## Methods

### Data sources and searches

This meta-analysis was performed according to the Preferred Reporting Items for Systematic Reviews and Meta-Analyses 2020 statement, an updated guideline for reporting systematic reviews (11) and was registered with INPLASY (INPLASY202320083). Systematic literature searches were conducted in the PubMed, EMBASE, Cochrane Library, and Web of Science databases from their inception to May 18, 2023. Variations of the terms ‘Heart’ or ‘Cardiac’ were combined with variants of the terms ‘Hypothyroidism’ and ‘Levothyroxine’ (the search strategy is shown in **Table 1**). All the references included in the study were traced back.

**Table 1:** Search strategy for PubMed and Embase.

Database	
PubMed	"hypothyroidism"[MeSH] OR "hypothyr*"[Title/Abstract] OR "thyroid deficien*"[Title/Abstract] OR "thyroid insufficien*"[Title/Abstract] AND "thyroid hormones"[MeSH] OR "thyroid hormon*"[Title/Abstract] OR "Levothyro*" [Title/Abstract] OR "Levothroid" [Title/Abstract] OR "Levoxine" [Title/Abstract] OR "Eltroxine" [Title/Abstract] OR "Euthyrox" [Title/Abstract] OR "Eutirox" [Title/Abstract] OR "thyronin*"[Title/Abstract] OR "thyroxin*"[Title/Abstract] OR "tyroxin*"[Title/Abstract] OR "L-T3"[Title/Abstract] OR "L-T4"[Title/Abstract] OR "FT3"[Title/Abstract] OR "FT4"[Title/Abstract] OR "t3 hormon*"[Title/Abstract] OR "t4 hormon*"[Title/Abstract] OR "T3"[Title/Abstract] OR "T4"[Title/Abstract] OR "substitution therap*"[Title/Abstract] AND "Echocardiography"[MeSH Terms] OR "stroke volume"[MeSH] OR "ventricular function "[MeSH] OR "diastole"[MeSH] OR "atrial remodeling"[MeSH] OR "Echocardiography"[Title/Abstract] OR "ventricular remodeling"[Title/Abstract] OR "cardiac remodeling"[Title/Abstract] OR "cardiac adaptation"[Title/Abstract] OR "lv geometry"[Title/Abstract] OR "left ventricular geometry"[Title/Abstract] OR "cardiac geometry"[Title/Abstract] OR "cardiac dimension"[Title/Abstract] OR "left ventricular function"[Title/Abstract] OR "systolic function"[Title/Abstract] OR "ejection fraction"[Title/Abstract] OR "diastolic function"[Title/Abstract] OR "atrial remodeling"[Title/Abstract] OR " strain "[Title/Abstract] 548 hits (18-05-2023)
Embase	'thyroid hormone'/exp OR ‘hypothyr*’:ab,ti OR ‘thyroid deficien*’:ab,ti OR ‘thyroid insufficien*’:ab,ti AND 'hypothyroidism'/exp OR 'thyroid hormon*':ab,ti OR 'levothyro*':ab,ti OR 'levothroid':ab,ti OR 'levoxine':ab,ti OR 'eltroxine':ab,ti OR 'euthyrox':ab,ti OR 'eutirox':ab,ti OR 'thyronin*':ab,ti OR 'thyroxin*':ab,ti OR 'tyroxin*':ab,ti OR 'l-t3':ab,ti OR 'l-t4':ab,ti OR 'ft3':ab,ti OR 'ft4':ab,ti OR 't3 hormon*':ab,ti OR 't4 hormon*':ab,ti OR 't3':ab,ti OR 't4':ab,ti OR 'substitution therap*':ab,ti AND 'echocardiography'/exp OR 'heart ventricle remodeling'/exp OR 'heart stroke volume'/exp OR 'heart function'/exp OR 'heart atrium remodeling'/exp OR ‘Echocardiography’:ab,ti OR ‘ventricular remodeling’:ab,ti OR ‘cardiac remodeling’:ab,ti OR ‘cardiac adaptation’:ab,ti OR ‘lv geometry’:ab,ti OR ‘left ventricular geometry’:ab,ti OR ‘cardiac geometry’:ab,ti OR ‘cardiac dimension’:ab,ti OR ‘left ventricular function’:ab,ti OR ‘systolic function’:ab,ti OR ‘ejection fraction’:ab,ti OR ‘diastolic function’:ab,ti OR ‘atrial remodeling’:ab,ti OR ' strain ':ab,ti 5067 hits (18-05-2023)

### Inclusion and exclusion criteria

All potential studies were identified by reading the titles and abstracts. Studies that were deemed suitable for inclusion were selected. The full text was then read further to determine the final inclusion criteria and exclude irrelevant literature.

The inclusion criteria for each study were as follows. First, the clinical outcomes of each study must have been continuous variables that describe the cardiac structure or function which were obtained using echocardiography. Second, the mean and SD of each outcome must have been available as values from baseline and after treatment or as the amount of change after treatment. Third, all studies must have been conducted in adults with SCH rather than in patients with specific diseases or special populations (e.g., children or pregnant women). Fourth, the authors explicitly mentioned that previously elevated serum thyrotropin levels returned to normal after treatment.

**Figure 1:**
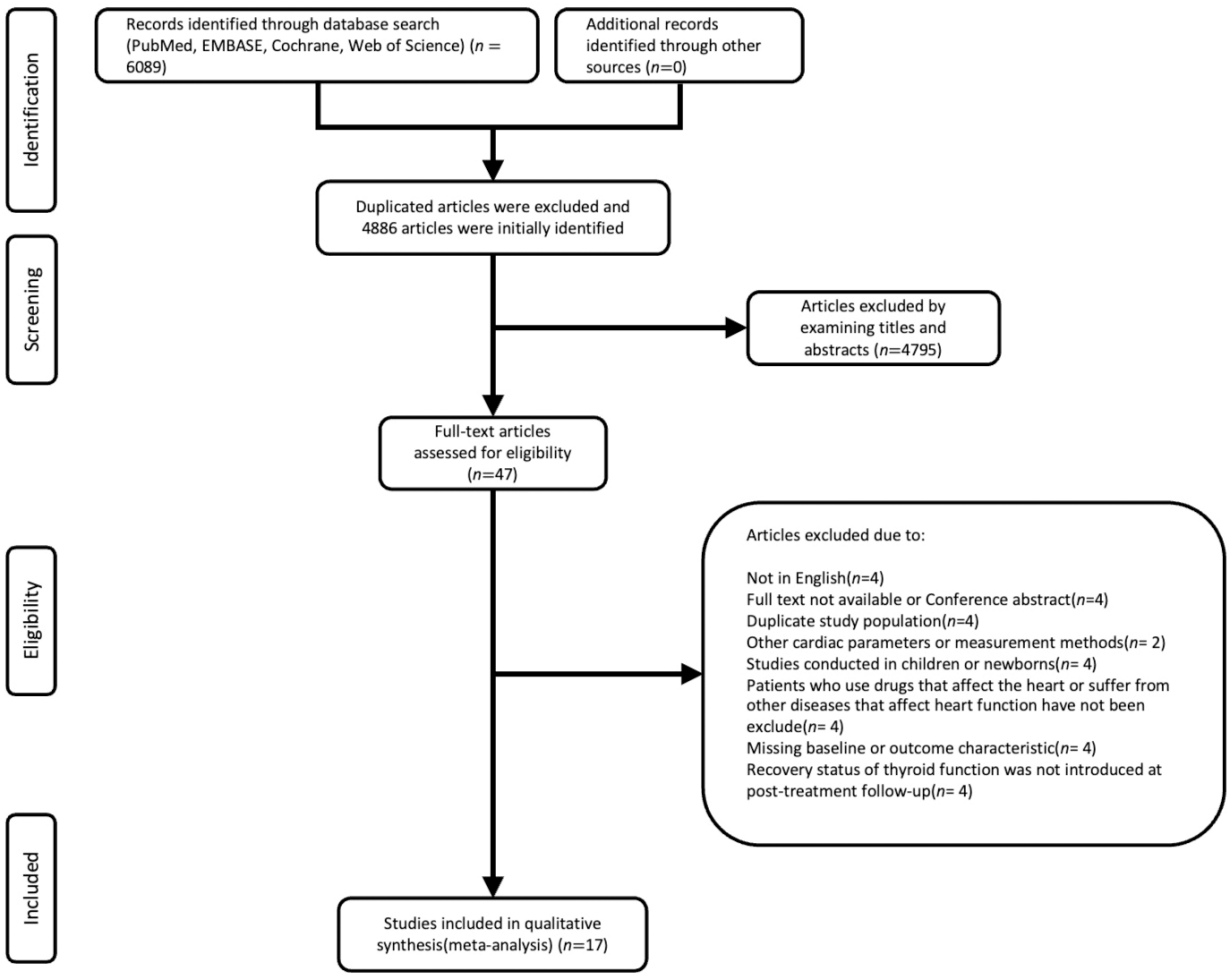
The general process of literature searching.

Studies were excluded if they met any of the following criteria: first, participants taking medications related to thyroid dysfunction (amiodarone, thyroid hormone replacement, and/or antithyroid drugs); second, subjects with comorbidities that could affect cardiac metrics, such as serious cardiovascular disease, hypertension, pulmonary heart disease, or diabetes mellitus; third, studies which focused on other unfavorable outcomes, such as right ventricular function and left atrial strain; fourth, studies which used methods other than echocardiography to measure cardiac function. The language used was restricted to English. Reviews, letters to the editor, case reports, and case series without follow-ups were excluded. The screening criteria are shown in **Figure 1**.

### Ultrasound measurements

Cardiac function was classified into three categories: cardiac morphology, diastolic function, and ventricular strain.

### Quality assessment

The Newcastle–Ottawa Scale (NOS), with a total of eight items, was chosen for the quality assessment of non-randomized research. The NOS includes study-specific criteria. Four of these items were used to score the selection of patients with SCH (four points). To assess the comparability of patients with SCH before and after therapy, one question was used (two points). The availability of cardiac outcomes was evaluated using three elements (three points). Finally, only studies with at least five points were included in the quantitative analysis. Following the screening process, all 17 candidate papers met the requirements and were used for further analysis, as indicated in **Table 2**. Two reviewers rated the included studies separately, and differences were resolved by consensus. No study was omitted based on the risk of bias assessment. Publication bias was visually examined using funnel plots.

**Table 2:** The Newcastle–Ottawa Scale for the assessment of the quality of studies.

Number	Study	Selection			Comparability		Exposure			Scores	
		Adequate definition of cases	Representativeness of the cases	Selection of controls	Definition of controls		Ascertainment of exposure	Same method	No-respons rate		
1	R.Arem 1996	1		1	1	1	1	1	1	8
2	Bernadette 1999	1		1	1	1	1	1	1	7
3	Chen 2016	1	1	1	1	1	1	1	1	8
4	Fabio 2001	1		1	1	1	1	1	1	7
5	Franzonia 2006	1		1	1	1	1	1	1	7
6	Huseyin 2006	1		1	1	1	1	1	1	7
7	Gulbanu 2011	1	1	1	1	1	1	1	1	8
8	Marijana 2014	1	1	1	1	1	1	1	1	8
9	Mehmet 2004	1		1	1	1	1	1	1	7
10	Milena 2021	1		1	1	1	1	1	1	7
11	Sahar 2021	1	1	1	1	1	1	1	1	8
12	Shatynska 2016	1		1	1	1	1	1	1	7
13	Tanase 2014	1		1	1	1	1	1	1	7
14	Valentina 2018	1	1	1	1	1	1	1	1	8
15	Fatma 2011	1		1	1	1	1	1	1	7
16	Owen 2006	1		1	1	1	1	1	1	7
17	CAMCI 2022	1		1	1	1	1	1	1	7

#### Data reporting and statistical analysis

The means, SDs, and number of subjects for each echocardiographic index at baseline and after thyroid hormone supplementation were extracted from each study. For the meta-analysis, we calculated mean differences before and after treatment for ventricular wall thickness, left atrial diameter (LAD), left ventricular mass index (LVMi), the ratio of E-velocity to A-velocity (E/A), the ratio of the mitral inflow E wave to the tissue Doppler e’ wave (E/e’), and LV strain, using Review Manager version 5.4 (The Nordic Cochrane Centre, Cochrane Collaboration, Copenhagen, Denmark) with inverse variance weights. The heterogeneity among the studies was estimated with χ^2^ heterogeneity and the I^2^ test. The I^2^ statistic was calculated to assess the proportion of the total variation in the study estimates due to heterogeneity. If I^2^ was greater than 25%, a random-effects model was used, and a fixed-effects model was used if I^2^ was < 25%. Sensitivity analysis was performed by removing each study individually. A P-value of *p <*0.05 was considered statistically significant.

## Results

### Characteristics of studies

The search yielded 6089 records. After removing duplicates, 4886 records were screened for eligibility. The screening of titles and abstracts resulted in 47 potentially eligible studies, in which cardiac structure and function were measured before and after levothyroxine treatment. Two studies (12, 13) reported overall and cardiac function in the same patient population. As both outcome measures were relevant for this review, we included both studies, but handled them as a single study. There were also two articles (14, 15) that included individuals from the same sample with overlap; we chose the one (15) with the highest number of participants. In total, 17 studies (9, 13, 15–29) and 568 patients with SCH met the inclusion criteria and were included in this review (**Figure 1**).

Most patients with SCH included in the study were female and were all over 18 years old. The duration of follow-up varied across studies, ranging from 1 to 12 months. The characteristics of the studies are listed in **Table 3**.

**Table 3:** characteristics of included studies.

Number	Study	Country	No. patient	Final participants	Mean age	Gender(M/F)	Thyrotropin threshold	TSH(before therepy)	TSH(atfer therepy)	Treatment duration
1	R.Arem 1996	American	8	8	36.4±6.02	6/2	>5.5mIU/L	14.8±9.5	3.0±1.6	3 months
2	Bernadette 1999	Italy	26	10	36 ± 12	2/24	>3mU/L	8.6±4.8	━━	6 months
3	Chen 2016	China	32	32	40.7±9.7	12/20	>4.2uIU/ml	5.62±1.34	3.86±1.07	12months
4	Fabio 2001	Italy	20	10	32.6±12.1	2/18	>3.6mIU/l	5.44±2.41	1.32±0.47	6 months
5	Franzonia 2006	Italy	42	16	52.2±15.1	13/29	>3.6mIU/l	8.8±1.7	━━	6 months
6	Huseyin 2006	Turkey	22	22	48±13	7/15	4.0ng/ml	13.3±9.1	━━	105±60days
7	Gulbanu 2011	Turkey	22	22	18-60	22F	4.2uIU/ml-9.9uIU/ml	7.17±1.74	2.28±0.63	3 months
8	Marijana 2014	Serbia	54	54	41±6	54F	>4mIU/L	8.8±2.7	2.15±0.7	1years
9	Mehmet 2004	Turkey	45	23	40.2±9.3	4/19	>4.0mU/L	8.47±1.9	3.34±1.7	6 months
10	Milena 2021	Serbia	35	35	51.6±15.4	6/29	4mIU/L-10mIU/L	6.9±2.1	━━	3 months
11	Sahar 2021	Egypt	36	36	40.2±8.6	4/32	>4.5IU/L	11(10-7)	2.2(1.9-2.4)	6 months
12	Shatynska 2016	Ukraine	33	33	51.21±4.32	9/24	>4.0mU/L	11.82±0.56	━━	6 months
13	Tanase 2014	Romania	75	75	60.86±12.56	8/67	>4.2 mUI/L	5.80±1.50	3.11 ± 0.56	6 months
14	Valentina 2018	Macedonia	54	54	43.1±12.4	2/52	>4.2mU/L	8.1±1.3	2.8±2.6	5 months
15	Fatma 2011	Turkey	27	27	35.4±11.4	3/24	>4.2mIU/mL	7.01±2.36	3.16±0.33	1~1.5 months
16	Owen 2006	England	19	19	49.2±3.8	19F	>5.5mU/L	8.8(5.7-21.6)	1.3(0.4-2.8)	6 months
17	CAMCI 2022	Turkey	70	70	44.1 ± 9.4	34/33	>4.0 mIU/L	7.95 ± 1.44	2.28 ± 0.7	6 months

#### Effects of levothyroxine treatment on Cardiac Morphology in SCH Patients

**Figure 2:**
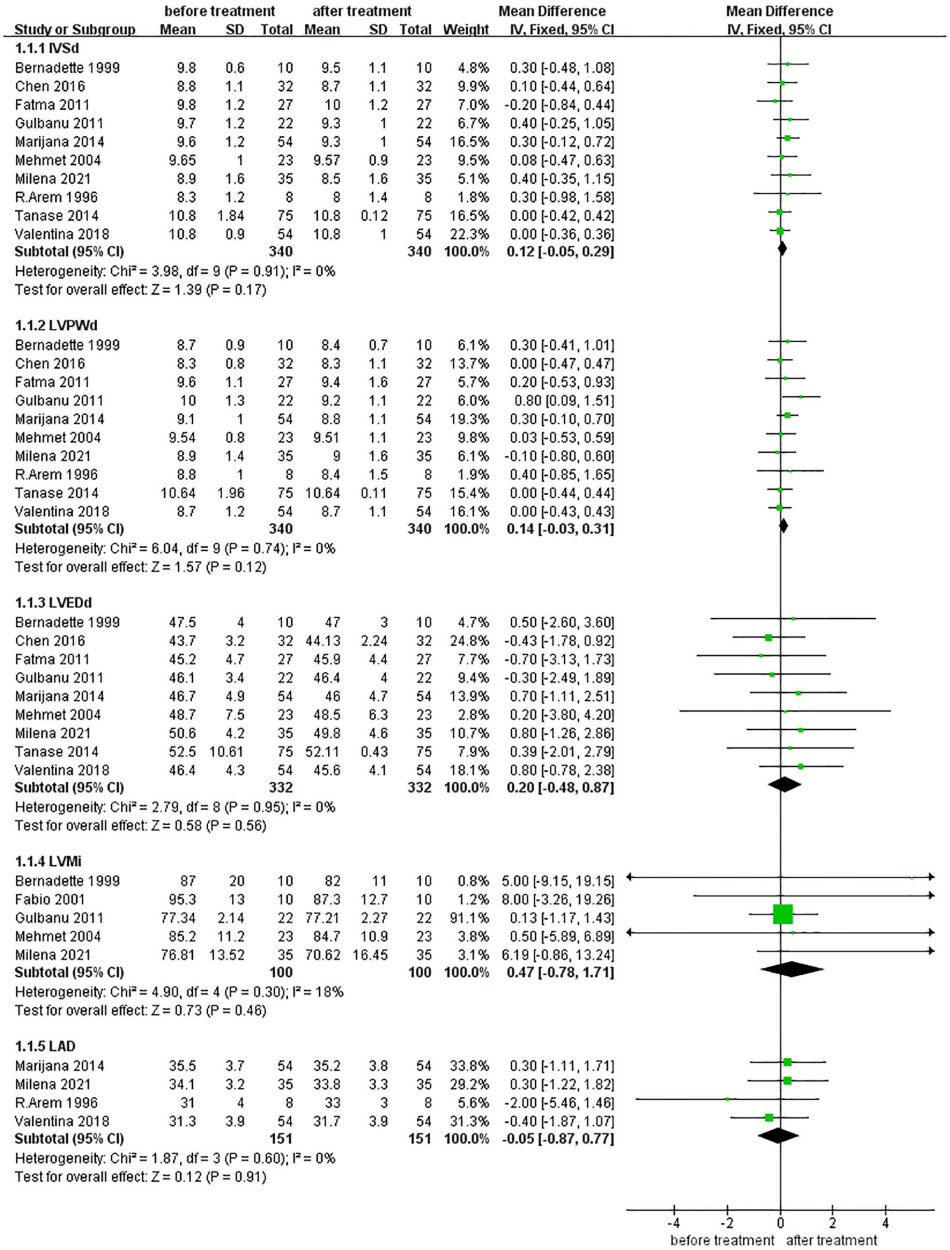
Comparison of cardiac morphology before and after levothyroxine treatment for SCH. IVSd, Interventricular Septal Thickness at Diastole: LVPWd, left ventricular posterior wall Thickness at Diastole; LVEDd, left ventricular end diastolic dimensions; LVMi, Left Ventricular mass index: LAD, left atrial diameter. LV mass index is the result of dividing the heart mass by the body surface area (g/m2).

As shown in **Figure 2**, five metrics for assessing the cardiac morphology were included in the quantitative analysis. These indicators included interventricular septal thickness at diastole (IVSd), left ventricular posterior wall thickness at diastole (LVPWd), left ventricular end-diastolic diameter (LVEDd), LVMi, and LAD. According to the results, there were no statistically significant differences between the five indicators before and after treatment (MD for IVSd 0.12 [95% CI, -0.05–0.29], *p* =0.17; MD for LVPWd 0.14 [95% CI, -0.03–0.31], *p* =0.12; MD for LVEDd 0.20 [95% CI, -0.48–0.87], *p* =0.57; MD for LVMi 0.47 [95%CI, -0.78, 1.71], *p*=0.46; MD for LAD -0.05 [95% CI, -0.87–0.77], *p* =0.91).

#### Effects of levothyroxine treatment on diastolic function in SCH Patients

**Figure 3:**
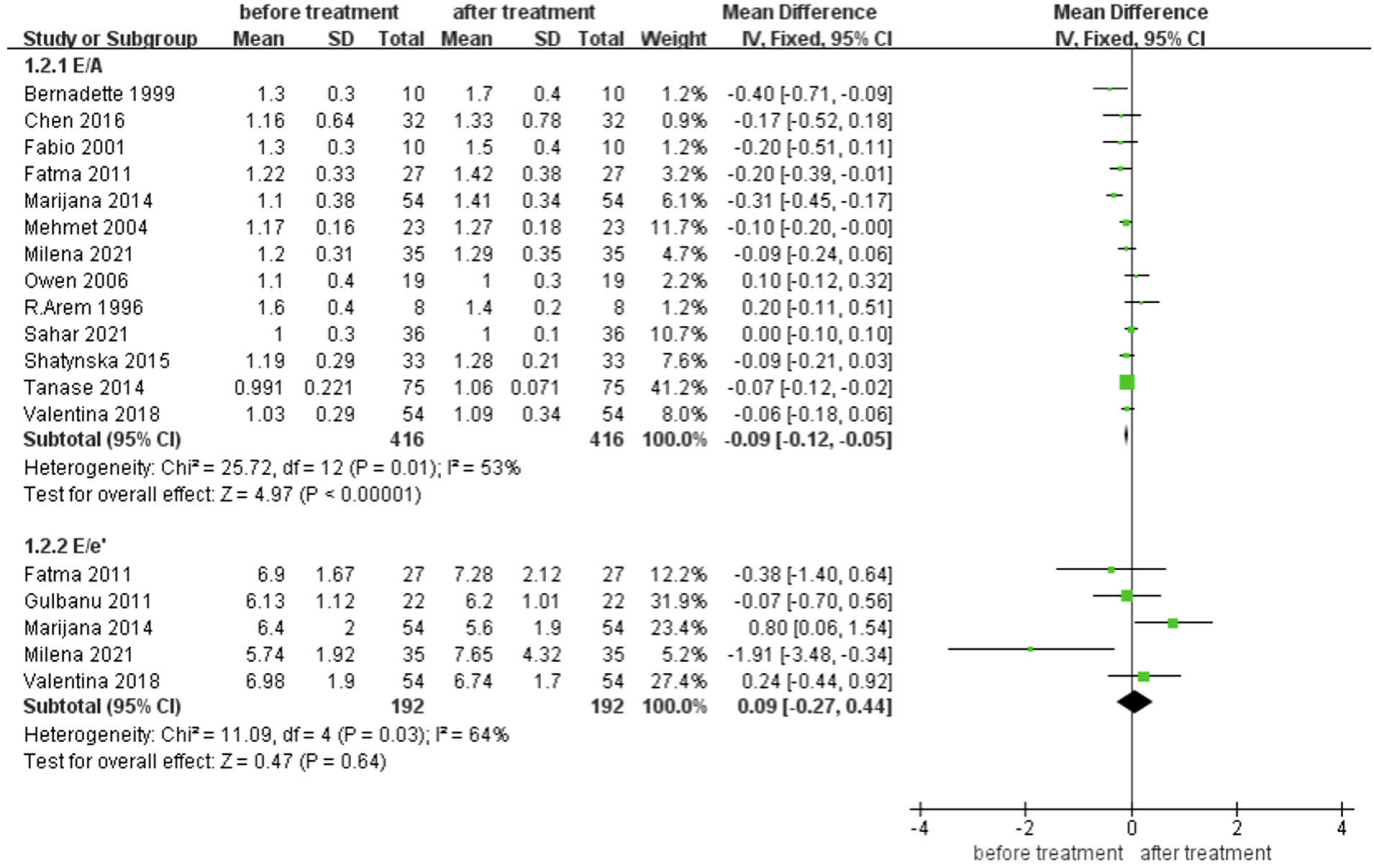
Comparison of Left ventricular diastolic function before and after levothyroxine treatment for SCH. E/A, the ratio of Left ventricular early diastolic mitral valve blood flow spectrum to the peak velocity of mitral valve blood flow formed by LA contraction in late diastole of LV. E/e’ the ratio of the mitral inflow E wave to the tissue Doppler e’ wave.

**Figure 3** includes two indicators of LV diastolic function. After treatment with levothyroxine, E/A significantly improved (MD for E/A -0.09 [95% CI, -0.12– -0.05], *p <*0.001), while E/e’ did not significantly improve (MD for E/e’ -0.09 [95% CI, -0.27–0.44], *p* =0.64).

#### Effects of levothyroxine treatment on myocardial strain in SCH Patients

**Figure 4:**
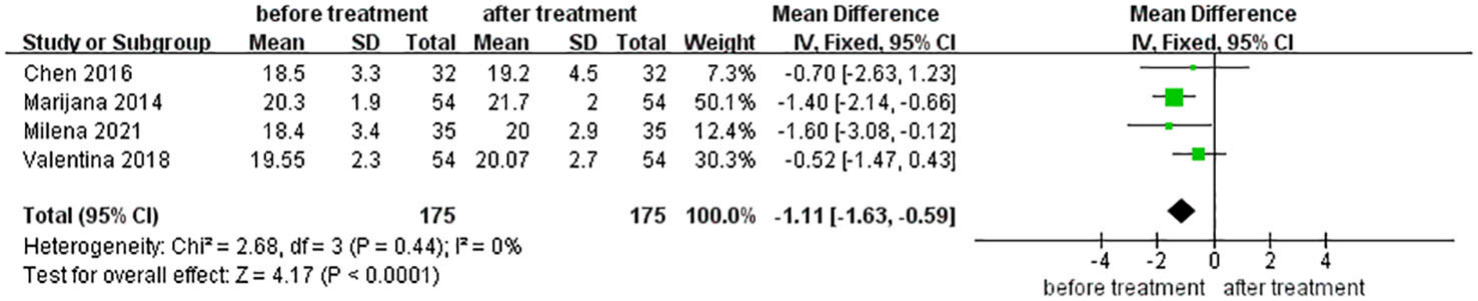
Comparison of Left ventricular Global longitudinal strain (GLS) before and after levothyroxine treatment for SCH.

GLS showed significant improvement after levothyroxine treatment (MD for GLS -1.11 [95% CI, -1.63– -0.59], *p <*0.001) (**Figure 4**).

## Discussion

This meta-analysis provided a comprehensive overview of the effects of levothyroxine on cardiac diastolic function in patients with SCH, assessed by echocardiography, based on 17 studies. Compared with before therapy, structural cardiac indices showed no significant changes after levothyroxine treatment in patients with SCH. In terms of diastolic function, although E/A improved, E/e’ did not improve after treatment. In our study, GLS also improved after levothyroxine treatment.

Our study showed that the IVSd, LVPWd, LVEDd, and LVMi in patients with SCH did not change significantly after treatment with levothyroxine. These LV structural indicators also represent LV remodeling. This result is consistent with that of another systematic evaluation based on echocardiography, cardiac magnetic resonance imaging, and myocardial radionuclide imaging, which found no significant morphological changes in the heart after treatment with levothyroxine in patients with SCH (30). Some studies have suggested that the LV mass of both adult and pediatric patients with SCH is significantly increased compared to that in the normal population (14, 31) and that this increases with the duration of the disease among those with TSH≥10.0 (32). However, other studies have reported no significant association between SCH and LV mass (33). Therefore, we consider that there may be two possible reasons: first, the TSH level of the patients in our study was mildly increased, so the LVMi at baseline may not have changed significantly compared with the normal population; second, the treatment time may have been insufficient to allow for significant changes in LV morphology.

In addition to LV thickness and mass, another vital structural index used to evaluate LV diastolic function was the LAD. However, this meta-analysis found no significant changes in LAD before and after levothyroxine treatment. The anteroposterior diameter of the left atrium is a commonly used clinical index to measure its size; however, a two-dimensional index cannot fully reflect the true size of the left atrium. At present, the left atrial volume index (LAVi) is a more accurate measurement index. Malhotra et al. (6) found that increased LAVi could reverse levothyroxine therapy in a population with LVDD. This study included 67 patients with SCH without underlying heart disease. After echocardiographic analysis, eight patients with LVDD were selected and treated with levothyroxine for 6 months. Their LAVi decreased from 31.17 ± 3.257 to 26.98 ± 2.668 (*p* =0.013). However, Nakova et al. (14) found that treatment with levothyroxine in average patients with SCH did not improve LAVi. We speculate that levothyroxine does not change the size of the left atrium in patients with SCH without LVDD.

For diastolic function, we found that E/A ratio increased after treatment with levothyroxine, but E/e’ did not change. E/A is a traditional index that has been widely used to evaluate LV relaxation, LV diastolic stiffness, and LV filling pressures (34). The E/A ratio was significantly lower in patients with SCH than in controls (6). Hormone replacement therapy in patients with SCH can increase the early diastolic left atrioventricular pressure gradient and improve the LV diastolic pattern. E/e’ is a commonly used index to evaluate LV diastolic function, LV filling pressure, and LA pressure (35, 36). Compared with blood flow Doppler, tissue Doppler is less load-dependent and has a minimal effect on LV filling pressure (37). However, there are few large evidence-based analyses using E/e’ to evaluate the effect of hormone replacement therapy in patients with SCH. According to a nested TRUST trial, diastolic heart function did not differ after treatment with levothyroxine compared to a placebo (10). However, in contrast to our study, participants in the TRUST trial were all aged ≥65 years, and functional indicators representing LV diastolic function other than E/e’ (such as E/A, E deceleration time) did not significantly improve. This may be due to the normal range of TSH increasing to higher values with aging (38). Some studies focusing on cardiovascular outcomes and cognitive function suggest that levothyroxine treatment is not beneficial in SCH patients aged 65 years or older (39, 40).

Our meta-analysis included a study by Tadic et al. (15) which differed from other included studies with their finding that E/e’ was significantly improved from baseline (6.4 ± 2 *VS.* 5.6 ± 1.9). In contrast to other studies, their observation duration was one year, rather than within six months. Therefore, further age-stratified randomized controlled studies and long-term observations are required to determine whether levothyroxine can improve E/e’.

Speckle tracking–based echocardiography (STE) has emerged as an interesting and promising tool for evaluating myocardial function. As LV diastolic and longitudinal systolic disturbances may stem from a common pathological background, they may develop in parallel. The presence of impaired GLS reinforces this diastolic dysfunction (41). GLS increased in patients with SCH after levothyroxine treatment. Although a decrease in GLS indicates impaired LV systolic function, an increasing number of studies have shown that it is related to diastolic function. Tschöpe and Senni found that GLS is a clinically and prognostically relevant parameter in patients with HFpEF (42). The 2019 Heart Failure Association (HFA) of the European Society of Cardiology (ESC) guidelines recommend including a cut-off point of 16% in absolute values as a secondary criterion for diagnosing HFpEF (43). Unlike tissue Doppler spot tracking, there are fewer sampling sites and fewer angle dependences (44). The improvement in LV long-axis strain recorded by spot tracking can better explain the improvement in LV function in patients with SCH after treatment with levothyroxine.

This study had some limitations. First, most included patients were middle-aged women, and we could not perform a subgroup analysis based on age and sex. Therefore, we could not draw detailed conclusions from specific populations. Future studies should clarify specific conclusions for patients with SCH of different ages, sexes, and SCH severity. Second, the differential diagnosis of elevated serum thyrotropin levels has been reported in various studies. Therefore, the heterogeneity caused by these factors may have affected our conclusions. Third, there are currently limited research data or observation times for some key indicators that evaluate diastolic function, such as LAVi and E/e’. Therefore, further studies with prolonged follow-ups are required to confirm whether SCH-induced myocardial morphological changes can be reversed by levothyroxine supplementation. Fourth, our study only included cardiac diastolic functions measured by echocardiography in patients with SCH. We did not further explore the lipid levels, BNP levels, carotid intima-media thickness, and cardiovascular outcomes of SCH patients after treatment with levothyroxine. Large RCTs should be conducted to confirm whether levothyroxine supplementation improves cardiovascular outcomes in all adult patients.

## Conclusion

In this study, we investigated the changes in cardiac diastolic function after thyroid hormone replacement therapy in adults with SCH. We found that the LV structure did not change significantly after hormone replacement therapy. Some parameters representing LV diastolic function improved after treatment; however, other parameters did not show significant improvement. The results of this study suggest that whether the use of levothyroxine replacement therapy is beneficial for the improvement of cardiac diastolic function in adults with SCH, is still unknown. However, as only some parameters showed improvement, there is a need for future studies to confirm that the cardiovascular benefits of levothyroxine replacement therapy apply to different age groups of SCH patients.

We would like to thank Editage (www.editage.cn) for English language editing.

## Data availability statement

The original contributions presented in the study are included in the article/supplementary material. Further inquiries can be directed to the corresponding authors.

## Author contributions

GL: Conceptualization, Funding acquisition, Writing – original draft, Writing – review & editing. MR: Data curation, Investigation, Software, Writing – original draft. YD: Formal Analysis, Investigation, Supervision, Writing – review & editing. RZ: Investigation, Project administration, Writing – review & editing. YW: Methodology, Supervision, Writing – review & editing. YL: Investigation, Resources, Writing – review & editing. LQ: Writing – review & editing.

## References

[1] Biondi B Cappola AR Cooper DS . Subclinical hypothyroidism: A review. JAMA (2019) 322:153. doi: 10.1001/jama.2019.9052 31287527

[2] Bekkering GE Agoritsas T Lytvyn L Heen AF Feller M Moutzouri E . Thyroid hormones treatment for subclinical hypothyroidism: a clinical practice guideline. BMJ (2019) 365:l2006. doi: 10.1136/bmj.l2006 31088853

[3] Inoue K Ritz B Brent GA Ebrahimi R Rhee CM Leung AM . Association of subclinical hypothyroidism and cardiovascular disease with mortality. JAMA Netw Open (2020) 3:e1920745. doi: 10.1001/jamanetworkopen.2019.20745 32031647 PMC12064075

[4] Gencer B Collet T-H Bauer D Gussekloo J Cappola A Nanchen D . Subclinical thyroid dysfunction and the risk of heart failure events: an individual participant data analysis from six prospective cohorts. J Gen Intern Med (2012) 27:S311–. doi: 10.1161/CIRCULATIONAHA.112.096024 PMC3884576 22821943

[5] Chen X Zhang N Cai Y Shi J . Evaluation of left ventricular diastolic function using tissue Doppler echocardiography and conventional doppler echocardiography in patients with subclinical hypothyroidism aged <60 years: A meta-analysis. J Cardiol (2013) 61:8–15. doi: 10.1016/j.jjcc.2012.08.017 23084577

[6] Malhotra Y Kaushik RM Kaushik R . Echocardiographic evaluation of left ventricular diastolic dysfunction in subclinical hypothyroidism: A case-control study. Endocr Res (2017) 42:198–208. doi: 10.1080/07435800.2017.1292524 28287839

[7] Huang W-H Sung K-T Kuo J-Y Chen Y-J Huang C-T Chien S-C . Atrioventricular longitudinal mechanics using novel speckle-tracking improved risk stratification beyond baseline thyroid hormone in asymptomatic subclinical hypothyroidism. Circ Cardiovasc Imaging (2021) 14:e012433. doi: 10.1161/CIRCIMAGING.121.012433 34784240

[8] Bielecka-Dabrowa A Godoy B Suzuki T Banach M von Haehling S . Subclinical hypothyroidism and the development of heart failure: an overview of risk and effects on cardiac function. Clin Res Cardiol (2019) 108:225–33. doi: 10.1007/s00392-018-1340-1 30091084

[9] Biondi B Fazio S Palmieri E Carella C Panza N Cittadini A . Left ventricular diastolic dysfunction in patients with subclinical hypothyroidism. J Clin Endocrinol Metab (1999) 84:2064–7. doi: 10.1210/jcem.84.6.5733 10372711

[10] Gencer B Moutzouri E Blum M Feller M Collet T Delgiovane C . The impact of levothyroxine on cardiac function in older adults with mild subclinical hypothyroidism: a randomized clinical trial. Am J Med (2020) 133:848–856.e5. doi: 10.1016/j.amjmed.2020.01.018 32171774

[11] Page MJ McKenzie JE Bossuyt PM Boutron I Hoffmann TC Mulrow CD . The PRISMA 2020 statement: an updated guideline for reporting systematic reviews. BMJ (2021) 372:n71. doi: 10.1136/bmj.n71 33782057 PMC8005924

[12] Pandrc M Ristic A Kostovski V Milin-Lazovic J Milic N Ciric J . The role of echocardiography in monitoring the therapeutic effect of levothyroxine replacement therapy in subclinical hypothyroidism. Arch Biol Sci (2020) 72:137–46. doi: 10.2298/ABS191029007P

[13] Pandrc MS Ristić A Kostovski V Milin-Lazović J Ćirić J . Calculation of left ventricular volumes and systolic indices in monitoring the therapeutic effect of levothyroxine replacement therapy in subclinical hypothyroidism. Int J Clin Pract (2021) 75:e14577. doi: 10.1111/ijcp.14577 34174124

[14] Ilic S Tadic M Ivanovic B Caparevic Z Trbojevic B Celic V . Left and right ventricular structure and function in subclinical hypothyroidism: The effects of one-year levothyroxine treatment. Med Sci Monit (2013) 19:960–8. doi: 10.12659/MSM.889621 PMC3829699 24217559

[15] Tadic M Ilic S Kostic N Caparevic Z Celic V . Subclinical hypothyroidism and left ventricular mechanics: A three-dimensional speckle tracking study. J Clin Endocrinol Metab (2014) 99:307–14. doi: 10.1210/jc.2013-3107 24187401

[16] Çamci S Yilmaz E Yakarişik M . The effect of L-thyroxine treatment on ventricular dysfunction and pulmonary arterial stiffness in patients with subclinical hypothyroidism. Eur Rev Med Pharmacol Sci (2022) 26:7036–45. doi: 10.26355/eurrev_202210_29887 36263551

[17] Arem R Rokey R Kiefe C Escalante DA Rodriguez A . Cardiac systolic and diastolic function at rest and exercise in subclinical hypothyroidism: Effect of thyroid hormone therapy. Thyroid (1996) 6:397–402. doi: 10.1089/thy.1996.6.397 8936662

[18] Chen T Han Y Zhang B Xia L Dong Y . Estimation of left ventricular myocardial mechanics in patients with subclinical hypothyroidism using three-dimensional speckle tracking echocardiography. J Med Imaging Health Inform (2016) 6:470–6. doi: 10.1166/jmihi.2016.1705

[19] Monzani F Di Bello V Caraccio N Bertini A Giorgi D Giusti C . Effect of levothyroxine on cardiac function and structure in subclinical hypothyroidism: A double blind, placebo-controlled study. J Clin Endocrinol Metab (2001) 86:1110–5. doi: 10.1210/jc.86.3.1110 11238494

[20] Franzoni F Galetta F Fallahi P Tocchini L Merico G Braccini L . Effect of L-thyroxine treatment on left ventricular function in subclinical hypothyroidism. Biomed Pharmacother BioMed Pharmacother (2006) 60:431–6. doi: 10.1016/j.biopha.2006.07.010 16935462

[21] Arinc H Gunduz H Tamer A Seyfeli E Kanat M Ozhan H . Tissue Doppler echocardiography in evaluation of cardiac effects of subclinical hypothyroidism. Int J Cardiovasc Imaging (2006) 22:177–86. doi: 10.1007/s10554-005-9030-2 16265602

[22] Erkan G Erkan AF Cemri M Karaahmetoglu S Cesur M Cengel A . The evaluation of diastolic dysfunction with tissue Doppler echocardiography in women with subclinical hypothyroidism and the effect of L-thyroxine treatment on diastolic dysfunction: a pilot study. J Thyroid Res (2011) 2011:654304. doi: 10.4061/2011/654304 21860776 PMC3153938

[23] Yazici M Gorgulu S Sertbas Y Erbilen E Albayrak S Yildiz O . Effects of thyroxin therapy on cardiac function in patients with subclinical hypothyroidism: index of myocardial performance in the evaluation of left ventricular function. Int J Cardiol (2004) 95:135–43. doi: 10.1016/j.ijcard.2003.05.015 15193811

[24] El Hini SH Mahmoud YZ Saedii AA Mahmoud SS Amin MA Mahmoud SR . Angiopoietin-like proteins 3, 4 and 8 are linked to cardiovascular function in naïve sub-clinical and overt hypothyroid patients receiving levothyroxine therapy. Endocr Connect (2021) 10:1570–83. doi: 10.1530/EC-21-0398 PMC8679937 34739390

[25] Shatynska-Mytsyk I Rodrigo L Cioccocioppo R Petrovic D Lakusic N Compostella L . The impact of thyroid hormone replacement therapy on left ventricular diastolic function in patients with subclinical hypothyroidism. J Endocrinol Invest (2016) 39:709–13. doi: 10.1007/s40618-015-0262-2 25740068

[26] Tanase D Vulpoi C Ionescu S Ouatu A Ambarus V Arsenescu-Georgescu C . Effects of sub clinical and overt primary hypothyroidism on the cardiac function and their reversibility under treatment using tissue doppler echocardiography. Acta Endocrinol-Buchar (2014) 10:640–53. doi: 10.4183/aeb.2014.640

[27] Nakova V Krstevska B Kostovska E Vaskova O Ismail L . The effect of levothyroxine treatment on left ventricular function in subclinical hypothyroidism. Arch Endocrinol Metab (2018) 62:392–8. doi: 10.20945/2359-3997000000052 PMC10118736 30304103

[28] Oner F Yurdakul S Oner E Uzum A Erguney M . Evaluation of the effect of L-thyroxin therapy on cardiac functions by using novel tissue Doppler-derived indices in patients with subclinical hypothyroidism. Acta Cardiol (2011) 66:47–55. doi: 10.2143/AC.66.1.2064966 21446380

[29] Owen P Rajiv C Vinereanu D Mathew T Fraser A Lazarus J . Subclinical hypothyroidism, arterial stiffness, and myocardial reserve. J Clin Endocrinol Metab (2006) 91:2126–32. doi: 10.1210/jc.2005-2108 16537677

[30] Wang X Wang H Li Q Wang P Xing Y Zhang F . Effect of levothyroxine supplementation on the cardiac morphology and function in patients with subclinical hypothyroidism: A systematic review and meta-analysis. J Clin Endocrinol Metab (2022) 107:2674–83. doi: 10.1210/clinem/dgac417 35810404

[31] Akin A Unal E Yildirim R Ture M Balik H Haspolat Y . Left and right ventricular functions may be impaired in children diagnosed with subclinical hypothyroidism. Sci Rep (2020) 10:19711. doi: 10.1038/s41598-020-76327-4 33184320 PMC7661521

[32] Rodondi N Cappola A Cornuz J Vittinghoff E Robbins J Fried L . Subclinical thyroid dysfunction, cardiac function, and the risk of congestive heart failure: The cardiovascular health study. Thyroid (2007) 17:S73–4. doi: 10.1089/thy.2007.1519 PMC2874755 18804743

[33] Pearce EN Yang Q Benjamin EJ Aragam J Vasan RS . Thyroid function and left ventricular structure and function in the framingham heart study. Thyroid (2010) 20:369–73. doi: 10.1089/thy.2009.0272 PMC2867586 20210671

[34] Chang S-N Juang JJ-M Tsai C-T Ko J-T Lien W-P . A novel integrated score index of echocardiographic indices for the evaluation of left ventricular diastolic function. PloS One (2015) 10:e0142175. doi: 10.1371/journal.pone.0142175 26555598 PMC4640516

[35] Nagueh SF . Left ventricular diastolic function: understanding pathophysiology, diagnosis, and prognosis with echocardiography. JACC Cardiovasc Imaging (2020) 13:228–44. doi: 10.1016/j.jcmg.2018.10.038 30982669

[36] Lassen MCH Biering-Sørensen SR Olsen FJ Skaarup KG Tolstrup K Qasim AN . Ratio of transmitral early filling velocity to early diastolic strain rate predicts long-term risk of cardiovascular morbidity and mortality in the general population. Eur Heart J (2019) 40:518–25. doi: 10.1093/eurheartj/ehy164 29659790

[37] Nagueh SF Smiseth OA Appleton CP Byrd BF Dokainish H Edvardsen T . Recommendations for the evaluation of left ventricular diastolic function by echocardiography: an update from the american society of echocardiography and the European association of cardiovascular imaging. Eur Heart J – Cardiovasc Imaging (2016) 17:1321–60. doi: 10.1093/ehjci/jew082 27422899

[38] Ross DS . Treating hypothyroidism is not always easy: When to treat subclinical hypothyroidism, TSH goals in the elderly, and alternatives to levothyroxine monotherapy. J Intern Med (2022) 291:128–40. doi: 10.1111/joim.13410 34766382

[39] Zijlstra LE Jukema JW Westendorp RGJ Du Puy RS Poortvliet RKE Kearney PM . Levothyroxine treatment and cardiovascular outcomes in older people with subclinical hypothyroidism: pooled individual results of two randomised controlled trials. Front Endocrinol (2021) 12:674841. doi: 10.3389/fendo.2021.674841 PMC8173189 34093444

[40] Parle J Roberts L Wilson S Pattison H Roalfe A Haque MS . A randomized controlled trial of the effect of thyroxine replacement on cognitive function in community-living elderly subjects with subclinical hypothyroidism: the Birmingham Elderly Thyroid study. J Clin Endocrinol Metab (2010) 95:3623–32. doi: 10.1210/jc.2009-2571 20501682

[41] Kosmala W Marwick TH . Asymptomatic left ventricular diastolic dysfunction. JACC Cardiovasc Imaging (2020) 13:215–27. doi: 10.1016/j.jcmg.2018.10.039 31005530

[42] Tschöpe C Senni M . Usefulness and clinical relevance of left ventricular global longitudinal systolic strain in patients with heart failure with preserved ejection fraction. Heart Fail Rev (2020) 25:67–73. doi: 10.1007/s10741-019-09853-7 31489515

[43] Pieske B Tschöpe C de Boer RA Fraser AG Anker SD Donal E . How to diagnose heart failure with preserved ejection fraction: the HFA–PEFF diagnostic algorithm: a consensus recommendation from the Heart Failure Association (HFA) of the European Society of Cardiology (ESC). Eur Heart J (2020) 22:391–412. doi: 10.1093/eurheartj/ehz641 32133741

[44] 2013 ESC guidelines on the management of stable coronary artery disease . The Task Force on the management of stable coronary artery disease of the European Society of Cardiology. Rev Esp Cardiol Engl Ed (2014) 67:135. doi: 10.1016/j.rec.2013.11.008 24795113

